# Satisfaction with cosmetic outcomes of breast reconstruction: Investigations into the correlation between the patients’ Breast-Q outcome and the judgment of panels

**DOI:** 10.1016/j.jpra.2020.03.002

**Published:** 2020-03-12

**Authors:** Y. Eltahir, E. Bosma, N. Teixeira, P.M.N. Werker, G.H. de Bock

**Affiliations:** a Department of Plastic Surgery, University of Groningen, University Medical Center Groningen, Hanzeplein 1, BB81, 9700 RB Groningen, the Netherlands; b Department of Epidemiology, University of Groningen, University Medical Center Groningen, Groningen, the Netherlands

**Keywords:** Strasser score, Breast reconstruction, Breast-Q, QoL, Outcomes, Patient satisfaction

## Abstract

**Objectives:**

We aimed to determine the relation between breast reconstruction method, patient satisfaction, and surgeon reported cosmetic outcome among women who underwent breast reconstruction after mastectomy.

**Study Design:**

A cross-sectional study of patients treated between 2006 and 2010.

**Main Outcome:**

Women's satisfaction with cosmetic outcomes after breast reconstruction.

**Measures:**

Cosmetic outcomes were evaluated by (1) women using the Breast-Q to rate satisfaction with breasts outcomes, and (2) an independent panel using the Strasser score. The relationships between the Breast-Q rating, Strasser scores, and breast reconstruction methods, including laterality and timing, were evaluated by Mann–Whitney *U* tests, Spearman's rank correlations, and Wilcoxon signed-rank tests.

**Results:**

Ninety-four women were included. Patients were more satisfied with their breasts if they had undergone autologous, unilateral, or secondary breast reconstruction compared with those who underwent alloplastic, bilateral, or primary breast reconstruction (*p*-values 0.008, 0.011, and 0.001, respectively). The Strasser system did not reveal significant cosmetic differences, with all breast reconstructions graded as mediocre or poor.

**Conclusions:**

Patient satisfaction with breast outcomes, as measured by the Breast-Q, was described as mediocre or poorly reflected by the Strasser score. If doctors are to support patients to make informed decisions on the optimal method of breast reconstruction, we need a more sensitive, comprehensive tool reflecting patients’ cosmetic outcomes.

## Introduction

Mastectomy has many negative effects on a woman's body image^1^ and can result in psychological changes,^2^ with fatigue, sleep disturbance, and depression among the associated complaints.^3^ weight gain, metabolic derangements, and loss of cardiorespiratory fitness might occur.^4^ Half of all women who undergo mastectomy develop a negative self-image and experience negative changes in their sexuality.^1^ Besides the obvious concerns regarding their health, breast cancer survivors also have been found to worry about their appearance following mastectomy, which is undoubtedly a disfiguring operation.^5^ These issues have clear effects on social and sexual relationships.^5^

Though morbidity and mortality were once the main concerns of breast cancer surgery, aesthetic satisfaction, as evaluated by the doctor or the woman herself, are increasingly recognized as important goals of breast cancer surgery.^6^ Research has shown that women are generally satisfied with the cosmetic outcomes of their surgery^7^ and that both women and doctors are satisfied with the outcomes.^8^ However, these studies were limited by failures to include independent reviews of outcomes, which may potentially have led to bias. In another study, the views of participating women were measured with non-validated or self-designed questionnaires.^9^ A methodological flaw in the study by Hunt et al was that esthetic results were assessed during a telephonic interview and patients were only examined when possible,^7^ while Tzafetta et al. did so during a specifically organized clinical interview and examination.^8^ In addition to these limitations and differences, no studies have investigated the relationship between a woman's satisfaction with the outcome of breast reconstruction, measured with the Breast-Q, and her doctor's evaluation.

In this study, we aimed to determine the relationship between the breast reconstruction method, the patient's satisfaction, and to evaluate cosmetic outcome among women who underwent breast reconstruction after mastectomy. The cross–sectional study focused on a specific stage of the whole reconstruction, the stage matching with the time that the patient was asked to fill out a specific module of the Breast-Q.

## Patients and methods

### Study population

We performed a cross-sectional study of women who underwent breast reconstruction following mastectomy at our center, including all eligible women from a previous study.^10^ We only included those who underwent successful breast reconstruction between 2006 and 2010, those who had a good understanding of the Dutch language, and those who provided signed informed consent. The exclusion criteria were as follows: metastatic disease, severe illness, inability to complete the questionnaire, and failed breast reconstruction due to complications resulting in either flap or prosthesis loss. As compared to our previous study,^10^ we included two additional women who underwent alloplastic reconstruction. The study was approved by the medical ethics committee of our institution.

### Measurements and procedure

Patient satisfaction was measured by the Breast-Q and cosmetic outcomes were assessed by using the Strasser Grading System by an independent panel of laypeople and experts.^11^

#### The Breast-Q

The Breast-Q questionnaire was used to assess the effect of mastectomy and satisfaction with breast reconstruction on the quality of life from the patient's perspective. For the current analysis, we only used the Breast-Q scales for satisfaction with breasts, nipples, and overall outcome.^12^

#### The independent review panel

We organized a review panel with 12 members who were independent of the surgical reconstruction team. The panel comprised three plastic surgeons, one oncology surgeon, two breast nurses, and three female and three male laypersons.

#### The Strasser Grading System

The Strasser Grading System was applied to provide an objective and reproducible grading of the esthetic outcomes.^11^^,^^13^ The system includes five subscales that grade malposition, distortion, asymmetry, contour deformity, and scar on 16-point scales. When the result is perfect or no flaws are seen, the score is 0; 1 is the score in case of any noticeable flaw, 5 for an obvious flaw and 15 for an obvious and deforming flaw. All points are added to get a total score ranging from 0 to 75, with an overall score of 0 indicating a perfect result, 1–4 a good result, 5–14 a mediocre result, and 15 or more a poor result.

#### Photographs

The medical photographer of our department took photographs according to standardized guidelines introduced by Persichetti in 2007.^14^ A photograph set comprised one front view, two lateral views, and two oblique views. The photographs were added to PowerPoint^Ⓡ^ (Microsoft^Ⓡ^, Groningen, the Netherlands) creating a slide show with all efforts made to hide patients’ identities. The study aimed to correlate the esthetic outcome and the patient perceived quality of life at two BR time points. Hence, the photographs used, were the photographs taken at the same time that the Breast-Q was filled in by the patient, which was between 4 and 52 months after the first stage of breast reconstruction.

### Procedure

Before the photographic assessments were made, we provided information to the independent panel on how to use the Strasser Grading System. Each member of the panel was then individually shown photographs on a computer screen in a random order, and was asked to score them individually on an online survey. Obtained data were stored in an Excel^Ⓡ^ (Microsoft^Ⓡ^, Groningen, the Netherlands) spreadsheet and saved on a password-protected computer at our institution.

### Statistics

The characteristics of patients and their breast cancer, as well as the treatments received, were stratified by the type of breast reconstruction (autologous versus alloplastic). Women with a combined implant and flap reconstruction were considered to have undergone alloplastic reconstruction. The Strasser scores by panel members were pooled such that a single Strasser score and range was given for each patient. Median Breast-Q and Strasser scores, with ranges (min–max), were generated for each type of reconstruction (alloplastic versus autologous, primary versus secondary, and unilateral versus bilateral), and compared by Mann–Whitney *U* tests. The distribution of Strasser scores between professional and lay panel members and between male and female panel members were evaluated by Spearman's rank correlations. We performed all statistical analyses using IBM SPSS, Version 22.0 (IBM Corp., Armonk, NY, USA).

## Results

### Population characteristics

**Figure 1:**
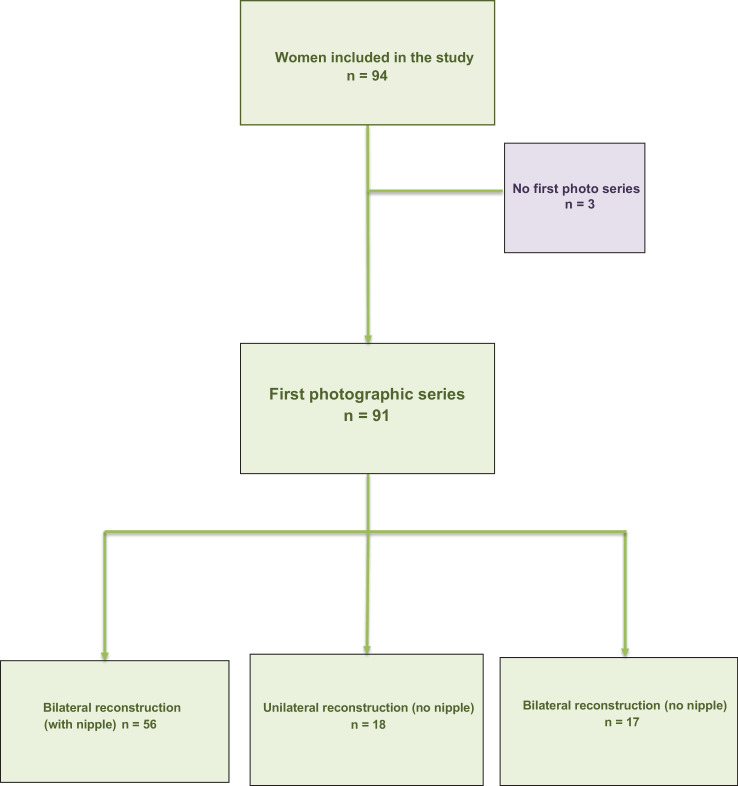
Flow Chart.

We enrolled 94 women in this study (Figure 1, Table 1), of whom 47 had undergone autologous and 47 had undergone alloplastic breast reconstructions. The average age at reconstruction was 44.4 years (range: 22–74 years). Concerning reconstruction type, there were 41 deep inferior epigastric perforator (DIEP) flaps, 34 breast implants, 12 implants plus latissimus dorsi muscle flaps, and 4 transverse musculocutaneous gracilis flaps. In addition, one patient underwent breast reconstruction using a free transverse rectus abdominis musculocutaneous (TRAM) flap, another patient in whom a DIEP flap on one side and a superficial inferior epigastric artery (SIEA) flap was performed, and one patient in whom an implant was combined with a SIEA flap.

**Table 1:** Characteristics of women by reconstruction type, n (%) or median (min–max).

Characteristics		Autologous n = 47	Implant n = 47
Age at mastectomy		45.0 (31–72)	42.0 (21–59)
Age at reconstruction		49.0 (31–74)	42.0 (22–59)
Age at questionnaires completed		51.0 (35–78)	44.0 (26–62)
Interval (months) between mastectomy and the first reconstruction		21.0 (0–135)	45.0 (0 to 90)
Interval (months) between the last reconstruction and questionnaire completion		26.0 (5–52)	23.5 (4–48)
Comorbidity *		11 (23.4%)	6 (12.8%)
BMI at time of surgery		26.0 (20–33)	23.0 (18–34)
BMI >30 kg/m^2^		10 (22.2%)	2 (4.3%)
Smoking		7 (14.9%)	14 (30.4%)
Chemotherapy		23 (48.9%)	13 (30.2%)
Radiotherapy		20 (42.6%)	6 (12.8%)
Mastectomy	Unilateral	33 (70.2%)	16 (34.0%)
	Bilateral	14 (29.8%)	31 (66.0%)
Reconstruction:	Unilateral	34 (72.3%)	15 (31.9%)
	Bilateral	13 (27.7%)	32 (68.1%)
TNM staging	Stadium 0–IIB	25 (71.4%)	23 (92.0%)
	Stadium IIIA–IIIC	10 (28.6%)	2 (8.0%)
BRCA1 or BRCA2 positive		9 (19.1%)	28 (59.6%)
Reconstruction:	Primary	17 (36.2%)	29 (61.7%)
	Secondary	30 (63.8%)	18 (38.3%)
Nipple reconstruction		32 (68.1%)	24 (51.1%)
Areola tattoo		26 (55.3%)	16 (34.0%)
Education:	Low	35 (74.5%)	22 (48.9%)
	High	12 (25.5%)	23 (51.1%)
Partner at time of questionnaire	Single	7 (15.2%)	7 (15.2%)
	Partner	39 (84.8%)	39 (84.8%)

Abbreviations: BMI = body mass index; BRCA1 = BRCA1, DNA repair associated gene; BRCA2 = BRCA2, DNA repair associated gene; TNM = TNM Classification of Malignant Tumors; data are condensed and divided into two categories: Stage 0 - IIB "and" Stage III – IIIC.

⁎ Including: diabetes mellitus, fibromyalgia, hypertension, and psychological instability.

### Photographs

Only 91 women had photographs taken, because 2 had undergone additional surgical treatment and 1 did not respond to our enquiries (Figure 1). Of the remaining women, 56 underwent bilateral breast and nipple reconstruction, 18 underwent unilateral breast reconstruction without nipple reconstruction, and 17 underwent bilateral breast reconstruction without nipple reconstruction.

### Breast-Q

The number of women per reconstruction, and their mean Breast-Q scores, is displayed in Table 2. The median Breast-Q scores indicate that the overall results were satisfactory. However, women were more satisfied with their breasts if they underwent autologous, unilateral, or secondary breast reconstructions when compared with alloplastic, bilateral, or primary breast reconstructions (p-values 0.008, 0.011, and 0.001, respectively).

**Table 2:** Comparison of satisfaction and quality overall, stratified for reconstruction type.

Breast Reconstruction	N	%	Breasts (*n* = 92) *	Outcome (*n* = 85) *	Nipples (*n* = 61) *	Strasser score ⁎⁎⁎ (*n* = 91) ⁎⁎
Total	92	100	70 (27–100)	75 (35–100)	67 (0–100)	11.0 (6.8–18.1)
Alloplastic	45	50	65 (27–100)	75 (35–100)	64 (0–100)	11.5 (6.8–16.2)
Autologous	47	50	77 (37–100)	86 (35–100)	67 (0–100)	10.8 (7.0–18.1)
P-value			0.008	0.089	0.912	0.409
Primary	45	50	65 (27–100)	67 (35–100)	64 (0–100)	9.7 (6.8–18.1)
Secondary	47	50	73 (37–100)	86 (55–100)	67 (0–100)	11.7 (8.0–15.8)
P-value			P = 0.080	P < 0.001	P = 0.452	P = 0.004
Unilateral	49	52.1	75 (34–100)	86 (47–100)	67 (0–100)	11.2 (7.4–16.2)
Bilateral	43	47.9	69 (27–100)	75 (35–100)	64 (0–100)	10.0 (6.8–18.1)
P-value			0.093	0.011	0.354	0.068

Results are given as median and range. P-values are based on Mann–Whitney *U* tests.

⁎ Breast-Q: Items refer to satisfaction with breast, outcome, and nipples on the Breast-Q. Not all women completed all Breast-Q questions.

⁎⁎ Strasser scores there was no Strasser score for three women.

⁎⁎⁎ The photographs were taken when women completed the Breast-Q

### Strasser scores

The details of the panel members and their scores are given in Table 3. The panel rated no breast reconstructions as perfect or good, 86 as mediocre, and 5 as poor. There were no differences in the distribution of Strasser scores between professionals and laypeople (*p* = 1.00) or between men and women (*p* = 0.81). Also, the Strasser scores were not significantly different when comparing alloplastic and autologous breast reconstructions or comparing bilateral and unilateral breast reconstructions (Table 2). However, the secondary breast reconstructions were associated with significantly better Strasser scores than the primary breast reconstructions (*p* = 0.004).

**Table 3:** Details of the panel members and their Strasser scores for the breast reconstructions.

Assessor	Sex	Age	Profession	Strasser score			
				Perfect	Good	Mediocre	Poor
1	Female	43	Administrative staff member	0	0	56	35
2	Female	46	Secretary	1	0	83	7
3	Male	53	Plastic surgeon	0	0	90	1
4	Male	25	Military	0	0	64	27
5	Male	29	Senior reporting and analysis	0	0	88	3
6	Female	23	Student	0	0	86	5
7	Female	40	Specialist breast nurse	0	0	84	7
8	Male	57	Oncological surgeon	0	0	74	17
9	Male	57	Specialist breast nurse	0	0	57	34
10	Male	25	Student	0	0	76	15
11	Male	44	Plastic surgeon	0	0	62	29
12	Male	39	Plastic surgeon	0	0	88	3

Scores were totalled and ranged from 0 to 75 per woman. Perfect, good, mediocre, and poor results were indicated by overall scores of 0, 1–4, 5–14, and ≥15, respectively.

### Breast-Q and Strasser scores

**Figure 2:**
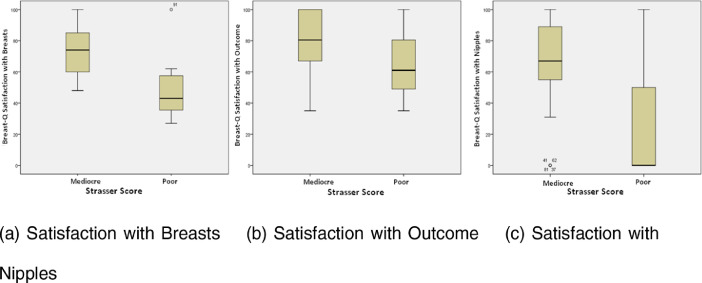
The correlation between Breast Q score and Strasser Score for the 3 Breast-Q scales.

The median Breast-Q scores and Strasser scores for each type of breast reconstruction are displayed in Table 4. There were correlations between poor Strasser scores and lower median scores for satisfaction with breasts (*p* < 0.001) and satisfaction with outcomes (*p* = 0.012). Only one patient reporting satisfaction with their nipples had a poor Strasser score. These correlations between the Strasser scores and the 3 Breast-Q scales “*Satisfaction with Breasts,*” “*Satisfaction with Outcome*” and “*Satisfaction with Nipples*” are displayed in Figure 2. There was a relation between the Breast-Q scale “Satisfaction with Breasts” and the esthetic score assessed with the Strasser System. Also, a similar relation was present between the “*Satisfaction with Outcome*” and the Strasser Score. Furthermore, there was no correlation between the Strasser Score and the Breast-Q scale “*Satisfaction with Nipples.*”

**Table 4:** Breast-Q scores stratified by Strasser scores (median; min – max).

Strasser score	N	%	Satisfaction with Breasts (*n* = 89)	Satisfaction with Outcome (n = 82)	Satisfaction with nipples (n = 59)
Mediocre	86	94.5	73 (45–100)	75 (35–100)	67 (0–100)
Poor	5	5.5	43 (34–53) *	61 (35–75) †	0 (0–0) ⁎⁎

⁎ p < 0.001

† p = 0.012. Not all women completed all Breast-Q questions or there was no Strasser score.

⁎⁎ Only 1 patient had a poor Strasser score.

## Discussion

Overall, we found that the differences in satisfaction among women, as reported by the Breast-Q, were not reflected by the Strasser scores. Indeed, although women were generally satisfied with their breasts and the outcomes of reconstruction, the independent panel evaluated all cosmetic outcomes as only mediocre or poor.

Of particular note, poor Strasser scores only correlated with lower median scores for satisfaction with breasts (*p* < 0.001) and satisfaction with outcomes (*p* = 0.012). Although this was disappointing, earlier research indicated a significant correlation between panel-rated and women-rated scores.^15^ However, women scored differently within the reconstruction subgroups, with the authors concluding that this was related to the sample size. Although the panel in that research found no cosmetic advantage for one type of reconstruction over another,^15^ our results are consistent with other research^10^^,^^16^^,^^17^ by showing that women were more satisfied with autologous than with alloplastic reconstruction (*p* = 0.008). Although women were more satisfied with their outcomes after unilateral than bilateral reconstruction (*p* = 0.011), the difference was not reflected by our panel's rating, which is similar to the results obtained previously using a four-point scale.^18^ One explanation for this finding is that breast symmetry is more important to patients, with asymmetry after breast-conserving surgery being significantly correlated with poor psychosocial outcomes.^19^ The inability of the Strasser score to reflect these differences should raise serious doubts about its sensitivity as a tool for measuring outcomes.

Women were more satisfied with their outcomes after secondary breast reconstruction than after primary breast reconstruction in this study (*p* < 0.001). This is logical, if we consider that half of all women are reported to experience a negative self-image and negative change in sexuality after mastectomy.^1^ This was also supported by the panel ratings, with secondary breast reconstructions associated with significantly better Strasser scores compared with primary breast reconstructions (*p* = 0.004). It was notable that many women chose not to complete the full reconstruction in this study, which is at odds with the findings of other research. For example, Elder et al. reported that the major determinant of esthetic satisfaction was procedure completion,^20^ while Wellisch et al. reported that nipple reconstruction improved overall satisfaction with breast reconstruction.^21^ However, our findings are consistent with those of Andrade et al., who showed no benefit to patient satisfaction from adding the reconstruction of the nipple–areola complex to breast mound revisions.^22^

**Figure 3:**
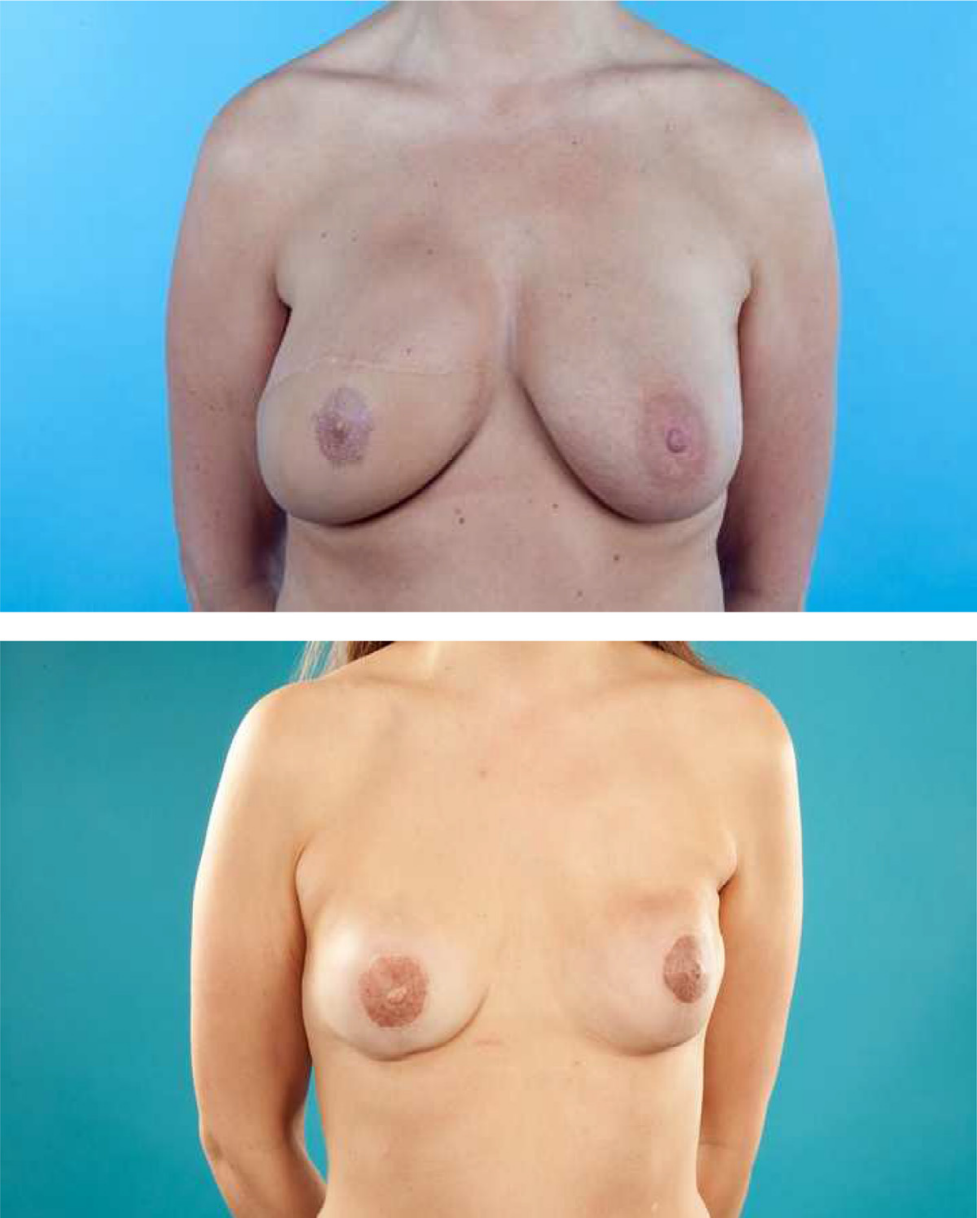
Both results were graded as “mediocre”.

Literature reviews indicate that no well-established, validated, or reproducible scoring systems exist for panels to use when rating esthetic outcomes after breast reconstruction, emphasizing the need for a reliable scale that can facilitate comparison.^23^^,^^24^ Potter et al. advised that size, shape, and symmetry be included in any esthetic evaluation,^23^ while Kim et al. recommended the need for an objective assessment of breast anatomy to improve the esthetic evaluation.^24^ Consistent with this argument, Ching et al. stated that the Strasser system had appropriate face and content validity, but that its validity and reliability had not been formally tested.^25^ Notwithstanding the lack of a validated and reproducible scoring system, and because of the downsides of the other scoring systems, we used the Strasser system because it was the best available option. As illustrated in Figure 3, however, we found this system to be rigid and unable to discriminate between some relevant differences.

We discovered that laypeople and professionals rated cosmetic results similarly using this system, which conflicts with earlier studies indicating that laypeople give harsher assessments, that women are more critical than men, and that surgical specialism can influence assessment.^26^, ^27^, ^28^ For example, Dian et al. reported that women and experts rated esthetic outcomes higher than laypersons.^29^ In other research, Cardoso et al. reported that the background of an assessor can affect their assessment, and that only experienced assessors should be allowed to assess esthetic outcomes because they had higher inter-rater agreement compared with inexperienced assessors.^27^^,^^30^ We disagree with this view because randomly selected laypeople are likely to be more representative of the patient's social network, a position supported by the fact that laypeople and professionals gave similar ratings in this study.

Furthermore, 3D imaging technologies are increasingly used particularly in cosmetic surgery, which helps the patient to get an idea of the expected result. In the future, such technology should be used more widely to provide a more objective prediction of the expected outcome. The study of Mailey et al.^31^ concluded that the 3D breast imaging system provides a highly reproducible 3D tool for measuring breast volume and simulating breast augmentation. Accuracy of the 3D models can vary up to 30%. Also, Oren et al.^32^ found that 3D analysis provides volumetric data that are of unique value for surgical planning and postoperative analysis. In the future, this development may result in a new approach and in studies comparing the satisfaction of women to computer esthetic outcome analysis.

The existing literature also leads to uncertainty as to whether women only, professionals only, or both should judge esthetic results. Some authors have stated that patient satisfaction is the most important parameter when evaluating esthetic outcomes,^33^^,^^34^ although it is accepted that this is influenced by the preoperative information they receive, their expectations, and their interaction with the surgeon.^35^ Indeed, when considering where the primacy of opinion lies, we should bear in mind the comment by Strasser, that “even a flawless result can be a bad result if the patient's desires are ignored”.^13^ Women's expectations, self-reported outcomes, and ultimately their satisfaction, should be considered the most important determinants of a successful cosmetic outcome from breast reconstruction, not our imposed standards of optimal outcome.

This study has limitations, including the fact that data were retrospective, that the time interval between breast reconstruction and the study was long for some women (2–3 years), the time span between photographs and the reconstructions varied between 1 and 52 months, and that not all women were at the same stage of breast reconstruction. Other limitations include the small sample size of the subgroups, the fact that data were from a single practice, and the fact that we grouped all autologous approaches together. The latter approach was chosen because we consider implants as the most important determinant of short- and long-term complications, irrespective of their precise coverage.

Furthermore, this study excluded women with failed reconstruction, due to the fact that their surgical results and psychosocial status do not match with any of our study groups.

However, grouping the surgical approaches and comparing the two main BR options, may be considered an advantage. In addition, our study benefits from having used patient-reported Breast-Q data. A final strength is that the panel represented both the lay and professional communities.

## Conclusion

In this study, the Strasser score only partially reflected women's satisfaction with reconstruction, as measured by the Breast-Q. Unfortunately, however, there are currently no better tools for understanding women's preferences or needs. Prospective research is therefore needed to design a more sensitive and comprehensive scoring system for cosmetic outcomes. It is our contention that women should be the final arbiters of preference, and that we need a comprehensive tool that reflects their preference at its heart. The information gained from this scoring system could help doctors to support patients when making informed decisions about the optimal method of breast reconstruction in a truly shared decision-making process.

## Declaration of Competing Interest

None
